# Supplementary material for: Effects of physical, chemical, and biological ageing on the mineralization of pine wood biochar by a Streptomyces isolate
Source: PLoS One. 2022 Apr 7;17(4):e0265663. doi: 10.1371/journal.pone.0265663 (PMC8989327; doi:10.1371/journal.pone.0265663)
Supplement: S1 Table — (DOCX) [file pone.0265663.s001.docx]

**S1 Table.** **FT-IR functional group peak assignment for biochar**

| Functional group | Wavenumber (cm^-1^) |
| --- | --- |
| O–H stretching of carboxylic acids, phenols, alcohols | 3370 [1,2] |
| Aliphatic C-H stretching | 2932 [1–3] |
| CO_2_ asymmetrical stretching | 2350 [4] |
| Carboxyl and carbonyl C=O stretching | 1701 [1–3] |
| Aromatic C=C vibrations | 1593 [1–3] |
| Aliphatic C-H deformation | 1413 [5,6] |
| *C–O stretching and O–H deformation of carboxylic groups and/or C–OH stretching of polysaccharides | 1200 [3,5] |
| Aromatic C-H out of plane deformation | 810 [7,8] |

*The peak at wavenumber 1200 cm^-1^ detected could be a combination of peaks observed in the region 1260-1200 cm^-1^: C–O stretching and O–H bending of COOH and 1170-950 cm^-1^: C–OH stretching of polysaccharides.
