# Supplementary material for: Effects of physical, chemical, and biological ageing on the mineralization of pine wood biochar by a Streptomyces isolate
Source: PLoS One. 2022 Apr 7;17(4):e0265663. doi: 10.1371/journal.pone.0265663 (PMC8989327; doi:10.1371/journal.pone.0265663)
Supplement: S2 Table — (DOCX) [file pone.0265663.s002.docx]

| **S2 Table.** FTIR spectra relative peak heights of the unaged and physically, chemically and biologically aged biochar samples produced at low temperature (350°C) and high temperature (550 °C) | | | | | | | |
| --- | --- | --- | --- | --- | --- | --- | --- |
| HTT (°C) | Ageing treatment | aromatic out of plane C-H stretch | C-O stretch and O-H bend in carboxylic acids* | aliphatic C-H stretch in CH_3_ and CH_2_ | aromatic C=C stretch in quinones | C=O stretch in carboxylic acids/ ketones | aliphatic C-H stretch in CH_3_ and CH_2_ |
|  |  | 810 cm^-1^ | 1200 cm^-1^ | 1413 cm^-1^ | 1593 cm^-1^ | 1701 cm^-1^ | 2932 cm^-1^ |
| 350 | Unaged | 0.09 | 0.29 | 0.12 | 0.36 | 0.08 | 0.07 |
|  | Physical | 0.05 | 0.32 | 0.00 | 0.28 | 0.35 | 0.00 |
|  | Chemical | 0.06 | 0.28 | 0.10 | 0.30 | 0.18 | 0.08 |
|  | Biological | 0.07 | 0.32 | 0.09 | 0.31 | 0.13 | 0.07 |
| 550 | Unaged | 0.28 | 0.19 | 0.02 | 0.41 | 0.09 | 0.01 |
|  | Physical | 0.12 | 0.23 | 0.00 | 0.42 | 0.17 | 0.05 |
|  | Chemical | 0.25 | 0.18 | 0.00 | 0.43 | 0.12 | 0.01 |
|  | Biological | 0.20 | 0.30 | 0.00 | 0.41 | 0.09 | 0.00 |
| *The peak at wavenumber 1200 cm^-1^ detected could be a combination of peaks observed in the region 1260-1200 cm^-1^: C–O stretching and O–H bending in carboxylic acids and 1170-950 cm^-1^: C–OH stretching in polysaccharides. | | | | | | | |
