# Supplementary material for: Effects of physical, chemical, and biological ageing on the mineralization of pine wood biochar by a Streptomyces isolate
Source: PLoS One. 2022 Apr 7;17(4):e0265663. doi: 10.1371/journal.pone.0265663 (PMC8989327; doi:10.1371/journal.pone.0265663)
Supplement: S1 Fig — (Left) Images of Streptomyces isolate growth on the surface of biochar nutrient agar media at the end of the incubation period for a replicate of (a) 350°C unaged biochar (b) 550°C unaged biochar (c) 350°C physically aged biochar (d) 550°C chemically aged biochar. (Right) Processed images of Streptomyces isolate growth on the surface of the biochar sample shown on the left using the ImageJ software. (DOCX) [file pone.0265663.s003.docx]

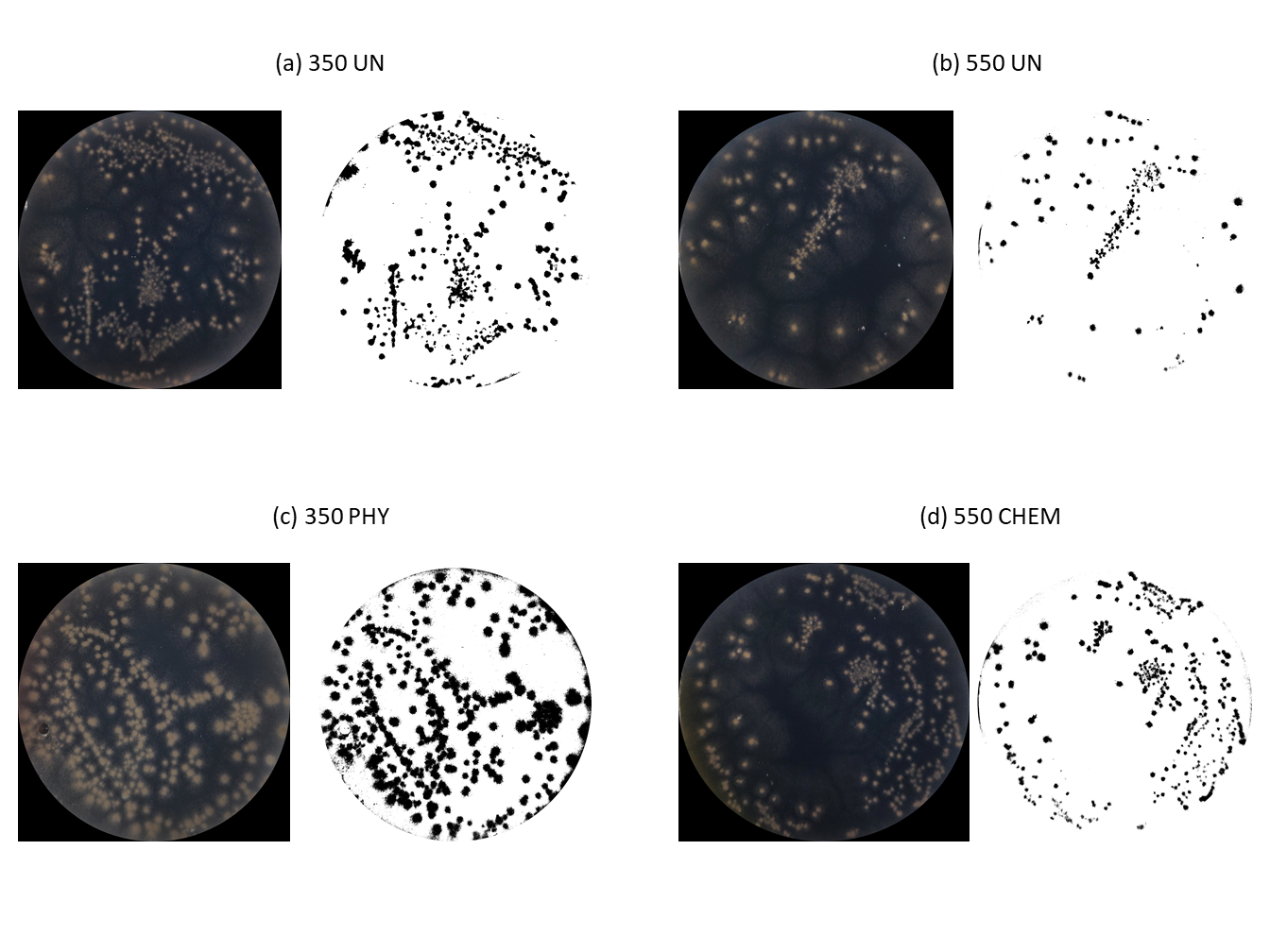
**S1 Fig.** **Images of Streptomyces isolate growth on biochar- raw and processed using ImageJ.** (Left) Images of *Streptomyces* isolate growth on the surface of biochar nutrient agar media at the end of the incubation period for a replicate of (a) 350 °C unaged biochar (b) 550 °C unaged biochar (c) 350 °C physically aged biochar (d) 550 °C chemically aged biochar. (Right) Processed images of *Streptomyces* isolate growth on the surface of the biochar sample shown on the left using the *Image J* software.
