# Supplementary figures and images for: Effects of physical, chemical, and biological ageing on the mineralization of pine wood biochar by a Streptomyces isolate
Source: PLoS One. 2022 Apr 7;17(4):e0265663. doi: 10.1371/journal.pone.0265663 (PMC8989327; doi:10.1371/journal.pone.0265663)

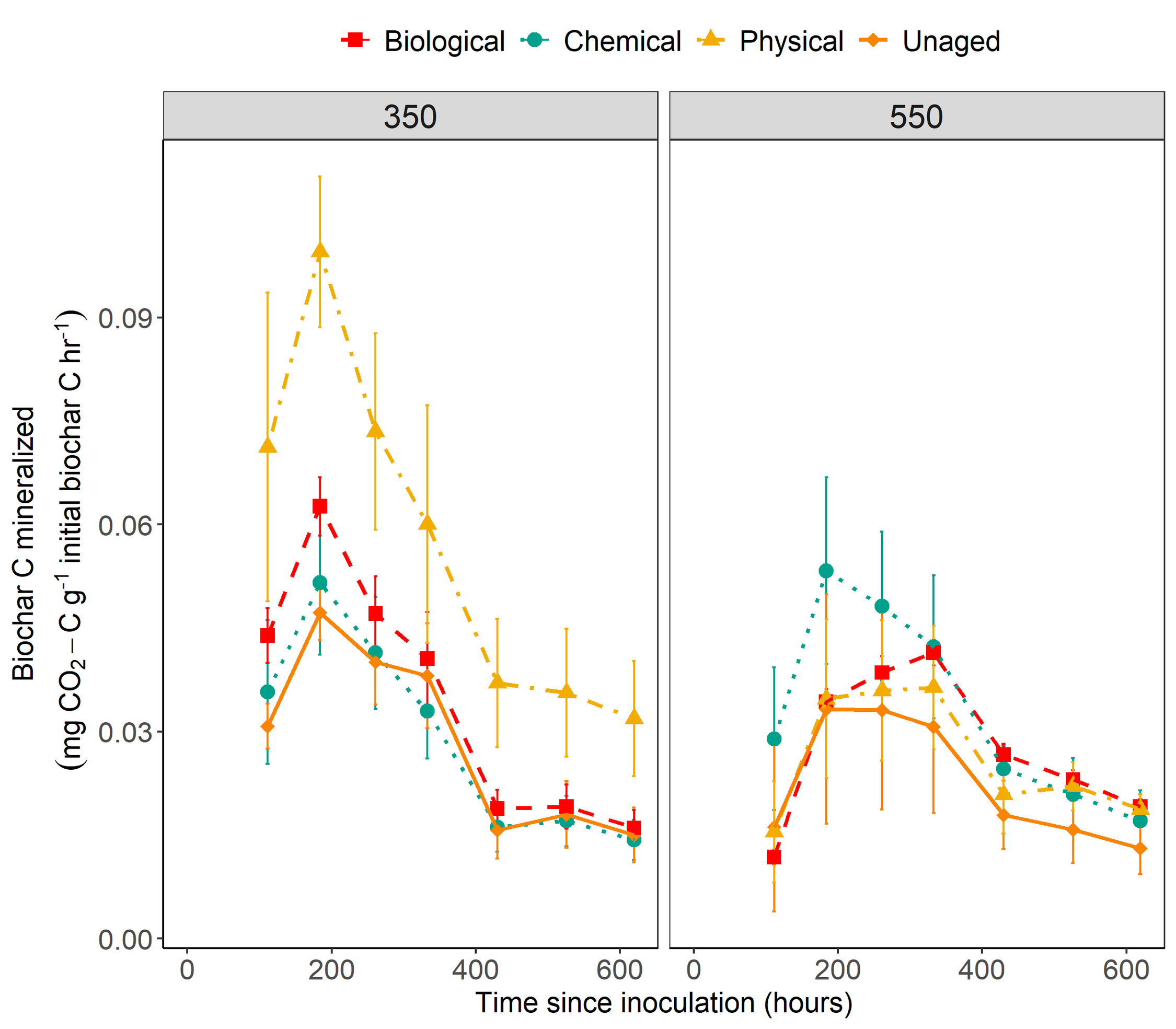

Supplement: S2 Fig — Data show mean C mineralization rate of unaged and physically, chemically and biologically aged biochar samples over time, with uninoculated blanks subtracted and normalized with mean biochar-C. N = 3 for physical, chemical and unaged, N = 5 for biological. Error bars represent 95% confidence intervals. The left panel shows biochar produced at 350°C and the right panel shows biochar produced at 550°C. (TIF) [file pone.0265663.s004.tif]

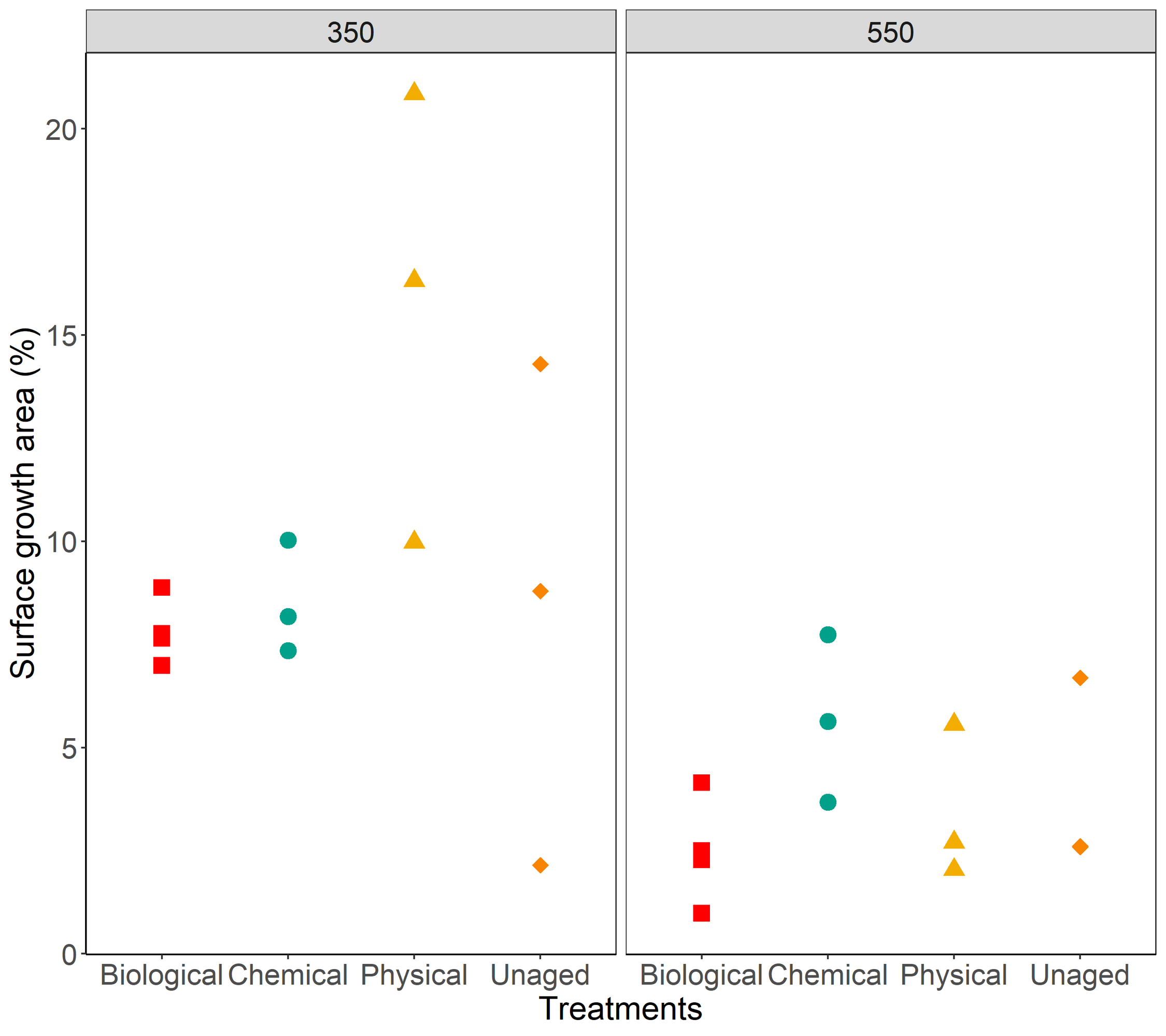

Supplement: S3 Fig — Growth of Streptomyces isolate on biologically, chemically, and physically, aged biochar and unaged biochar agar media over the incubation period. N = 3 for physical, chemical and unaged, N = 5 for biological. The left panel shows biochars produced at 350°C and the right panel shows biochars produced at 550°C. (TIF) [file pone.0265663.s005.tif]

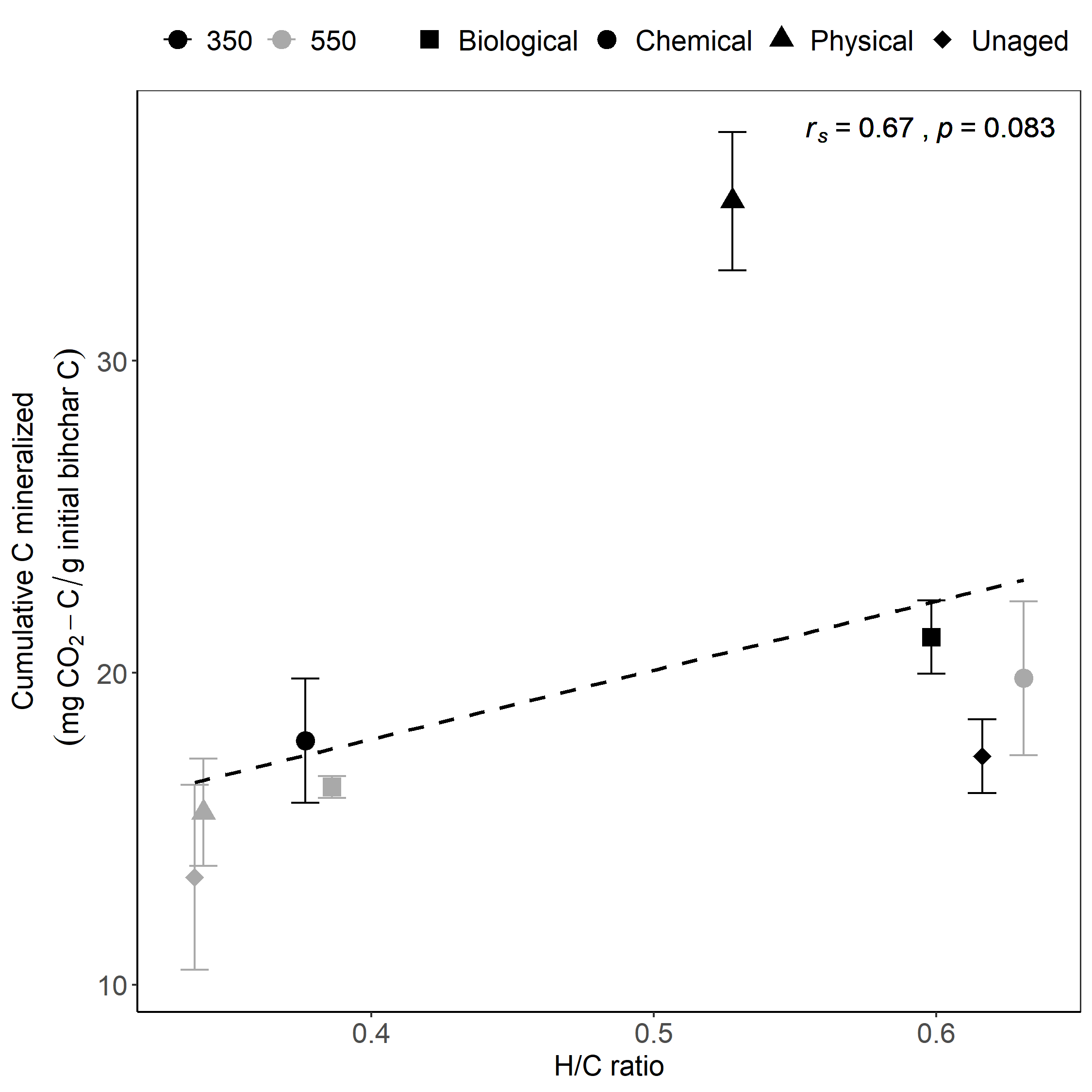

Supplement: S4 Fig — N = 3 for physical, chemical and unaged, N = 5 for biological treatments. Error bars represent standard error of the mean. Shapes indicate unaged, physically, chemically and biologically aged biochar samples produced at 350°C (black) and 550°C (gray). (TIF) [file pone.0265663.s006.tif]
