# Supplementary material for: Effects of physical, chemical, and biological ageing on the mineralization of pine wood biochar by a Streptomyces isolate
Source: PLoS One. 2022 Apr 7;17(4):e0265663. doi: 10.1371/journal.pone.0265663 (PMC8989327; doi:10.1371/journal.pone.0265663)
Supplement: S1 Appendix — (DOCX) [file pone.0265663.s007.docx]

S1 Appendix for:

Effects of physical, chemical, and biological ageing on the mineralization of pine-wood biochar by a *Streptomyces* isolate

Nayela Zeba, Timothy D. Berry, Kevin Panke-Buisse, and Thea L. Whitman

1. **Materials and Methods**

*Production of biochar*

Biochar was produced from eastern white pine wood chips (*Pinus strobus* (L.)) at 350 and 550 °C in a modified Fischer Scientific Lindberg/Blue M Moldatherm box furnace (Thermo Fisher Scientific, Waltham, MA, USA) fitted with an Omega CN9600 SERIES Autotune Temperature Controller (Omega Engineering Inc., Norwalk, CT, USA) [9]. The feedstock was placed in a steel cylinder inside the furnace chamber and subjected to a continuous argon gas supply at a rate of 1 L min^-1^ to maintain anaerobic conditions during pyrolysis. The heating rate for production of biochar was kept constant at 5 °C min^-1^ and mixing of the feedstock inside the steel cylinder via mechanical paddles started once temperatures hit 250 °C. We held the temperature constant for 30 minutes once the highest treatment temperature was reached (350 and 550 °C), after which the product was rapidly cooled by circulating cold water in stainless steel tubes wrapped around the steel cylinder. Pyrolyzed material was ground using a ball mill and sieved to collect biochar with particle size <45 µm.

*Chemical Analyses*

Elemental analysis: Total C and N were determined for aged and unaged biochar samples using a Thermo Scientific Flash EA 1112 Flash Combustion Analyzer (Thermo Fisher Scientific, Waltham, MA, USA) at the Department of Agronomy, UW- Madison, WI, USA. Total H was determined using a Thermo Delta V isotope ratio mass spectrometer interfaced to a Temperature Conversion Elemental Analyzer (Thermo Fisher Scientific, Waltham, MA, USA) at the Cornell Isotope Laboratory, NY, USA. In order to estimate total O by subtraction, we determined ash content of aged and unaged biochar samples using the method prescribed by ASTM D1762-84.

Replicates were included for all samples for the CHN elemental analysis and ash measurements. Total O was calculated by subtraction as per Enders et al. [10] as follows:

O (%w/w) = 100 - C (%w/w) - N (%w/w) - H (%w/w) - ash (%w/w)

pH: The pH of aged and unaged biochar samples was measured in deionized water at a 1:20 solid:solution ratio using an Inlab Micro Combination pH electrode (Mettler Toledo, Columbus, OH, USA) connected to a Thermo Scientific Orion Star A111 benchtop pH meter (Thermo Fisher Scientific, Waltham, MA, USA). Briefly, we added 0.15 g of biochar to 3 mL of deionized water in 5 mL centrifuge tubes and vortexed for 1 hour at low speed. After vortexing, the tubes were centrifuged at 15000 X g for 2 min to sediment the biochar particles. We aliquoted 200 μL of the clear suspension into microtubes and measured the pH. All pH measurements were performed in replicates except in the case of 350PHY and 550PHY where we had limited material.

Fourier-transform infrared (FT-IR) spectroscopy: We quantified the heights of selected functional groups using the Shimadzu IR Solution FT-IR software. The spectra were attenuated total reflection (ATR) corrected to approximate a transmission spectrum and smoothed to reduce the background noise. Spectra baselines were drawn in the following regions using a quartic fitting function: 3800-2300 cm^-1^, 2300-1820 cm^-1^, 1820-1500 cm^-1^, 1500-925 cm^-1^ and 925-700 cm^-1^. Wavenumbers were assigned for selected functional groups based on studies as described in Table S1. Peaks were manually detected around specific wavenumbers by identifying peak maxima, upper and lower baselines for each of the functional groups. Baselines for selected peaks were drawn as follows: 3006-2783 cm^-1^ for aliphatic C-H stretch, 1808-1645 cm^-1^ for C=O stretch, 1670-1483 cm^-1^ for C=C vibrations and stretch, 1501-1307 cm^-1^ for C-H bending of CH2 and CH3, 1334-919 cm^-1^ for C–O stretching, O–H bending of COOH and/or C–OH stretching of polysaccharides and 934-713 cm^-1^ for aromatic C-H deformation.

1. **Biochar nutrient media preparation**

The final biochar nutrient media used for biochar incubations is a combination of the following components:

1. Nutrient solution which consists of
   1. Basal salt solution (described in detail below)
   2. Trace elements solution (described in detail below)
   3. Vitamin mixture (described in detail below)
   4. Vitamin B12
2. Agar and biochar suspension

The same media recipe was used throughout the study to (i) isolate and recover the *Streptomyces* sp. on biochar (ii) perform the biological ageing treatment of biochar with a microbial community and (iii) perform the incubation study of the *Streptomyces* isolate with aged and unaged biochar. For the biological ageing treatment, we used the biochar suspension without addition of agar.

To prepare the final biochar nutrient media agar plates (per 1 L):

Agar and biochar suspension = 500 mL

Basal salt solution = 500 mL

Vitamin B12 = 200 uL of 250 mg L-1 solution* , **

Vitamin mix = 200 uL* , **

Trace elements solution = 1 mL* , **

*To be added after autoclaving

**Filter sterilize with 0.2 micron filter

To prepare the biochar agar suspension (per 1 L):

Nobel agar = 60 g

Ground biochar = 2 g

To prepare the basal salt solution (per 1 L):

KH_2_PO_4_ = 0.4 g

NH_4_Cl = 0.5 g

KCl = 1.0 g

CaCl_2_. 2H_2_O = 0.3 g

NaCl = 2 g

MgCl_2_. 6H_2_O = 1.24 g

NaSO_4_ = 5.86 g

Note: This solution will end up being acidic with a pH of ~6, The pH was adjusted to 7 by addition of NaoH and buffered by adding 1 g of MES (2-(N-Morpholino) ethane sulfonic acid) (VWR International, Radnor, PA, USA) before autoclaving.

To prepare the trace elements solution (aka SL-10, per 1 L):

HCl 25% (v/v) = 10 mL

FeCl_2_. 4H_2_0 = 1.5 g

CoCl_2_. 6H_2_0 = 190 mg

MnCl_2_. 4H_2_0 = 100 mg

ZnCl_2_ = 70 mg

H_3_BO_3_ = 6 mg

Na_2_MoO_4_. 2H_2_O = 36 mg

NiCl_2_. 6H_2_O = 24 mg

CuCl_2_. 2H_2_O = 2 mg

To prepare the vitamin mixture (per 1L):

4-aminobenzoic acid (Vitamin B9 precursor) = 40 mg

D(+)-biotin (Vitamin B7) = 10 mg

Nicotinamide (Vitaman B3) = 100 mg

D(+)-pantothetic acid hemicalcium (Vitamin B5) = 50 mg

Pyridoxamine dihydrocloride (Vitamin B6) = 100 mg

Thiamine dihydrochloride (Vitamin B1) = 100 mg

1. **Effect of pyrolysis temperature on biochar C mineralization of unaged biochar**

Mean cumulative biochar C mineralized at the end of the incubation was significantly higher by 39% for 350 °C biochars compared to 550 °C biochars (Kruskal-Wallis_ANOVA_, *p* value = 0.01). This is consistent with previous studies that have noted higher microbial activity and respiration in incubations with low temperature chars [15–18], since biochar produced at high pyrolysis temperature contains a larger fraction of condensed aromatic C, which is more difficult for microorganisms to oxidize [19–23]. Mean colony growth on agar surfaces over the month-long incubation, as measured by percentage of total surface area, was 166% higher for 350 °C biochar treatments as compared to their 550 °C counterparts (S3 Fig.). This corresponds to trends observed in the cumulative biochar C mineralized over the incubation period, indicating that the *Streptomyces* strain more effectively colonizes and grows on agar surfaces containing biochar particles produced at 350 °C.

1. **Effect of pyrolysis temperature on the elemental composition and surface chemistry of unaged biochar**

*Elemental analysis*

For unaged biochar, the total C in 550UN was higher (mean = 84.7%) than that in 350UN (mean = 74.8%), while the total O and H contents were lower in 550UN (mean O = 11.9%; mean H = 2.4%) compared to 350UN (mean O = 20.4%; mean H = 3.9%; Table 1). This is consistent with previous studies that have reported an increase in the total carbon content of biochars with increasing pyrolysis temperature, due to increased carbonization and greater relative loss of H and O [10,12].

*FTIR*

While comparing the individual FTIR peaks between 350UN and 550UN, we observed changes in regions associated with aliphatic and aromatic C groups (Fig. 2a). There was, however, no notable change in relative height for the C=O stretch peak in carboxylic acids from 350UN (0.08) to 550UN (0.09). For the aromatic C functional groups, we observed an increase in the relative peak height with increasing pyrolysis temperatures (Fig. 2a and S2 Table). The greatest increase appeared in the 810 cm^-1^ aromatic C-H out of plane deformation (0.09 to 0.28) and a slight increase was seen in the 1593 cm^−1^ C=C aromatic stretch (0.36 to 0.41). In contrast, the relative peak heights of groups indicative of aliphatic and lignin/cellulose-derived transformation products that are present in low temperature biochar decreased with increasing pyrolysis temperature. We observed a slight decrease in the relative peak height from 350UN to 550UN for 2932 cm^-1^ aliphatic C-H stretch of CH_3_ and CH_2_ (0.07 to 0.01), 1413 cm^‑1^ C-H bending of CH_3_ and CH_2_ (0.12 to 0.02) and 1200 cm^-1^ C–O stretching of phenols/ COOH/ polysaccharides (0.29 to 0.19). With the exception of the C=O carboxyl stretch, these broad trends in surface chemistry with increasing pyrolysis temperature are consistent with previous studies that investigated the effect of pyrolysis temperature on the chemical properties of biochar [19,24,25].

**Supplementary References**

1. Guo Y, Bustin RM. FTIR spectroscopy and reflectance of modern charcoals and fungal decayed woods: implications for studies of inertinite in coals. Int J Coal Geol. 1998;37: 29–53. doi:https://doi.org/10.1016/S0166-5162(98)00019-6

2. Cheng C-H, Lehmann J, Thies JE, Burton SD, Engelhard MH. Oxidation of black carbon by biotic and abiotic processes. Org Geochem. 2006;37: 1477–1488. doi:https://doi.org/10.1016/j.orggeochem.2006.06.022

3. Chen J, Gu B, Leboeuf EJ, Pan H, Dai S. Spectroscopic characterization of the structural and functional properties of natural organic matter fractions. Chemosphere. 2002;48: 59–68. doi:10.1016/s0045-6535(02)00041-3

4. Chatterjee R, Sajjadi B, Chen W-Y, Mattern DL, Hammer N, Raman V, et al. Effect of Pyrolysis Temperature on PhysicoChemical Properties and Acoustic-Based Amination of Biochar for Efficient CO2 Adsorption. Front Energy Res. 2020;8: 85. doi:10.3389/fenrg.2020.00085

5. Solomon D, Lehmann J, Kinyangi J, Liang B, Schäfer T. Carbon K-Edge NEXAFS and FTIR-ATR spectroscopic investigation of organic carbon speciation in soils. Soil Sci Soc Am J. 2005;69: 107–119. doi:https://doi.org/10.2136/sssaj2005.0107dup

6. Stevenson FJ. Humus Chemistry: Genesis, Composition, Reactions. 2nd ed. New York: John Wiley & Sons Inc.; 1994.

7. Politou AS, Morterra C, Low MJD. Infrared studies of carbons. XII The formation of chars from a polycarbonate. Carbon N Y. 1990;28: 529–538. doi:https://doi.org/10.1016/0008-6223(90)90049-5

8. Dutta S, Brocke R, Hartkopf-Fröder C, Littke R, Wilkes H, Mann U. Highly aromatic character of biogeomacromolecules in Chitinozoa: A spectroscopic and pyrolytic study. Org Geochem. 2007;38: 1625–1642. doi:https://doi.org/10.1016/j.orggeochem.2007.06.014

9. Güereña DT, Lehmann J, Thies JE, Enders A, Karanja N, Neufeldt H. Partitioning the contributions of biochar properties to enhanced biological nitrogen fixation in common bean (Phaseolus vulgaris). Biol Fertil soils. 2015;51: 479–491. doi:10.1007/s00374-014-0990-z

10. Enders A, Hanley K, Whitman T, Joseph S, Lehmann J. Characterization of biochars to evaluate recalcitrance and agronomic performance. Bioresour Technol. 2012;114: 644–653. doi:10.1016/j.biortech.2012.03.022

11. Mukherjee A, Zimmerman AR, Harris W. Surface chemistry variations among a series of laboratory-produced biochars. Geoderma. 2011;163: 247–255. doi:https://doi.org/10.1016/j.geoderma.2011.04.021

12. Ronsse F, van Hecke S, Dickinson D, Prins W. Production and characterization of slow pyrolysis biochar: influence of feedstock type and pyrolysis conditions. GCB Bioenergy. 2013;5: 104–115. doi:https://doi.org/10.1111/gcbb.12018

13. Huff MD, Lee JW. Biochar-surface oxygenation with hydrogen peroxide. J Environ Manage. 2016;165: 17–21. doi:10.1016/j.jenvman.2015.08.046

14. Mukherjee A, Zimmerman AR, Hamdan R, Cooper WT. Physicochemical changes in pyrogenic organic matter (biochar) after 15 months of field aging. Solid Earth. 2014;5: 693–704. doi:10.5194/se-5-693-2014

15. Dai Z, Barberán A, Li Y, Brookes PC, Xu J. Bacterial community composition associated with pyrogenic organic matter (biochar) varies with pyrolysis temperature and colonization environment. mSphere. 2017;2: e00085-17. doi:10.1128/mSphere.00085-17

16. Zimmerman AR, Gao B, Ahn M-Y. Positive and negative carbon mineralization priming effects among a variety of biochar-amended soils. Soil Biol Biochem. 2011;43: 1169–1179. doi:10.1016/j.soilbio.2011.02.005

17. Luo Y, Durenkamp M, De Nobili M, Lin Q, Brookes PC. Short term soil priming effects and the mineralisation of biochar following its incorporation to soils of different pH. Soil Biol Biochem. 2011;43: 2304–2314. doi:https://doi.org/10.1016/j.soilbio.2011.07.020

18. Bruun E, Hauggaard-Nielsen H, Ibrahim N, Egsgaard H, Ambus P, Jensen P, et al. Influence of fast pyrolysis temperature on biochar labile fraction and short-term carbon loss in a loamy soil. Biomass Bioenergy. 2011;35: 1182–1189. doi:10.1016/j.biombioe.2010.12.008

19. Keiluweit M, Nico PS, Johnson MG, Kleber M. Dynamic molecular structure of plant biomass-derived black carbon (biochar). Environ Sci Technol. 2010;44: 1247–1253. doi:10.1021/es9031419

20. Wiedemeier DB, Abiven S, Hockaday WC, Keiluweit M, Kleber M, Masiello CA, et al. Aromaticity and degree of aromatic condensation of char. Org Geochem. 2015;78: 135–143. doi:https://doi.org/10.1016/j.orggeochem.2014.10.002

21. Singh BP, Cowie AL, Smernik RJ. Biochar carbon stability in a clayey soil as a function of feedstock and pyrolysis temperature. Environ Sci Technol. 2012;46: 11770–11778. doi:10.1021/es302545b

22. Wang T, Camps Arbestain M, Hedley M. Predicting C aromaticity of biochars based on their elemental composition. Org Geochem. 2013;62: 1–6. doi:10.1016/j.orggeochem.2013.06.012

23. Harvey OR, Kuo L-J, Zimmerman AR, Louchouarn P, Amonette JE, Herbert BE. An nndex-based approach to assessing recalcitrance and soil carbon sequestration potential of engineered black carbons (biochars). Environ Sci Technol. 2012;46: 1415–1421. doi:10.1021/es2040398

24. Nguyen BT, Lehmann J, Hockaday WC, Joseph S, Masiello CA. Temperature sensitivity of black carbon decomposition and oxidation. Environ Sci Technol. 2010;44: 3324–3331. doi:10.1021/es903016y

25. Singh B, Fang Y, Johnston CT. A Fourier-Transform Infrared study of biochar aging in soils. Soil Sci Soc Am J. 2016;80: 613–622. doi:10.2136/sssaj2015.11.0414
